# Selection of the critical effect size alters hazard characterization – a retrospective analysis of key studies used for risk assessments of PFAS

**DOI:** 10.3389/ftox.2025.1525089

**Published:** 2025-03-14

**Authors:** L. Brunken, A. Vieira Silva, M. Öberg

**Affiliations:** Unit of Integrative Toxicology, Institute of Environmental Medicine, Karolinska Institutet, Stockholm, Sweden

**Keywords:** benchmark dose modeling, critical effect size, per-and polyfluoroalkyl substances, PFAS, risk assessment, Bayesian statistics, benchmark response, guidance values

## Abstract

Regulatory values for per- and polyfluoroalkyl substances (PFAS) vary widely across agencies, creating inconsistencies that challenge effective risk management and public health communication. These differences often stem from methodological choices in determining points of departure (PoDs), the selection of critical effect size (CES) and the modeling framework for benchmark dose (BMD) analysis. This study investigates the impact of CES selection on hazard characterization by analyzing how variations in CES influence resulting PoDs and health-based guidance values. A retrospective analysis of key studies from four regulatory PFAS risk assessments was conducted, covering both animal and epidemiological data (thyroid hormone, cholesterol, and vaccine response). CES options compared included 5%, 10%, one standard deviation from background, and a generalized effect size theory, using both frequentist and Bayesian statistics. The findings show that CES selection and statistical approach substantially affect BMD estimates such as the lower bound BMD (BMDL) of the respective confidence interval or credible interval; with larger CES values and Bayesian modeling yielding more biologically relevant, stable results. For instance, Bayesian methods provided narrower credible intervals, compared to frequentist methods at lower CES levels, minimizing overly conservative assessments. However, in comparison to the PoD previously derived by the European Food Safety Authority the results generally suggest lower values. In conclusion, this study supports the use of a flexible, endpoint-specific CES with Bayesian model averaging, which may enhance the accuracy and consistency of PFAS guidance values, offering a more robust foundation for regulatory risk assessments.

## 1 Background

Health-based guidance values (HBGVs) are essential for assessing the potential risks associated with chemical exposure in various regulatory contexts, including, but not limited to, food and drinking water. The regulations of per- and polyfluoroalkyl substances (PFAS) are recent, unfortunate examples of reference doses and guidance values diverging by several orders of magnitude (Cordner et al., 2019; Reinikainen et al., 2024). For oral intake, the European Food Safety Authority (EFSA) has derived a tolerable weekly intake (TWI) level of 4.4 ng/kg body weight (b.w.) per week for the sum of four PFAS (perfluorooctane sulfonic acid (PFOS), perfluorooctanoic acid (PFOA), perfluorohexane sulfonic acid (PFHxS) and perfluorononanoic acid (PFNA)) (EFSA CONTAM Panel, 2020). The U.S. Agency for Toxic Substances and Disease Registry (US ATSDR) derived minimal risk levels (MRLs) of 2 ng/kg b.w. per day for PFOS, 20 ng/kg b.w. per day for PFHxS and 3 ng/kg b.w. per day for PFOA and PFNA (ATSDR, 2021). Another example of divergence between reference doses originates from the legal framework of the European Drinking Water Directive (EU 2020/2184) of 2020, with its limit of 100 ng/L for the sum of 20 PFAS, which requires member states to set own maximum limits for PFAS (European Parliament and EU-Council, 2020). Some European countries have adopted stricter drinking water guidelines, including varying numbers of PFAS, (Denmark 2 ng/L; Sweden and Flanders 4 ng/L; the Netherlands 4.4 ng/L, Germany 20 ng/L, and Spain 70 ng/L) (Danish Ministry of Environment, 2021; European Environmental Bureau, 2023; German Federal Ministry of Health, 2023; Ministerio de la presidencia, 2023; RIVM, 2022; Livsmedelsverket, 2022; Vrancken, 2022). However, other EU countries have opposed such measures and have implemented only the directive’s minimum requirement (100 ng/L). The lack of international harmonization and inconsistent approaches among different programs and agencies hamper effective risk management and communication, especially when the risk assessment process lacks transparency at critical decision points.

Under the current paradigm of chemical health risk assessment, hazard characterization details the nature and extent of the identified adverse health effects (WHO, 2021). A key element of hazard characterization is the dose-response assessment, to identify a threshold below which no adverse effects are to be expected. The derivation of such a reference point should also include an uncertainty analysis to provide a clear understanding of the confidence in the point of departure (PoD, also known as reference point, RP) used in further quantification of risk (e.g., in the derivation of tolerable intake values).

In Europe, risk assessment is legally framed through measures such as EC No 1907/2006 (REACH) and the regulation (EU) 2023/915 (repealed regulation (EC) No 1881/2006), on setting maximum levels for certain contaminants in foodstuffs, conducted by agencies like the European Chemicals Agency (ECHA) and the EFSA (European Parliament and EU-Council, 2006; European Parliament and EU-Council, 2023). Most European countries also have independent competent authorities. In the United States, the process is defined by organizations such as the US EPA, with additional independent bodies in various states. Typically, the risk characterization begins with the identification of a critical effect and a critical/key study for this effect. The PoD is then determined through a dose-response analysis, that may be based on groupwise comparisons identifying the No Observed Adverse Effect Level, (NOAEL), or on dose-response modeling (using all data to identify the BMDL).

Previous studies have highlighted decision points that strongly influence the outcome of a risk assessment. The selection and interpretation of critical studies in deriving acute exposure guidance values (Öberg et al., 2010) and occupational exposure limits (Schenk et al., 2024), have been shown to be major causes of diverging guidance values. The method for deriving a PoD from a single study can vary substantially between risk-assessing bodies. Historically, the NOAEL was the most common approach, but the BMD method is now preferred by several major regulatory organizations, including the EFSA, the WHO and the US EPA (EFSA Scientific Committee, 2022; US EPA, 2012a; WHO, 2009).

The NOAEL approach has several identified limitations, such as relying on specific experimental doses and disregarding most dose-response information, potentially underestimating risk in lower-power studies (Bokkers and Slob, 2007; Crump, 1984; Dourson et al., 1985; EFSA Scientific Committee, 2017; Gaylor et al., 1998; Haber et al., 2018; Leisenring and Ryan, 1992; US EPA, 1995; US EPA, 2012b; Zarn et al., 2015). Conversely, the BMD approach, which is considered more robust and statistically advanced, determines a dose corresponding to a predefined response level (i.e., the critical effect size, CES), using the entire dose-response data range, allowing for interpolation between doses and for the quantification of uncertainty (Haber et al., 2018; Sand et al., 2008; Setzer and Kimmel, 2003). The BMD standard approach yields a 90% confidence interval (frequentist approach) or credible interval (Bayesian statistics), comprising a lower bound BMDL and upper bound BMDU. In a conservative approach aiming for protection, the BMDL typically serves as the PoD for subsequent guidance values (EFSA Scientific Committee, 2017; Slob, 2014; Slob, 2017).

For continuous data, such as the key effects measures used in PFAS risk assessment, the CES (also referred to as the benchmark response, BMR) is a pre-specified effect level important for describing the dose-response relationships and for the subsequent derivation of guidance values (EFSA Scientific Committee, 2017; Slob, 2002). Despite various concepts for CES selection, no universal framework exists. Two distinct types of metrics are in use to define the CES: those expressed as a relative (%) change compared to the background response level, and those based on the variation in the controls (e.g., the standard deviation, SD). For continuous datasets, a BMDL from a CES of 5% change in response over background has been shown to produce similar estimates to that of the NOAEL approach (Bokkers and Slob, 2005; Bokkers and Slob, 2007; Kavlock et al., 1995; Kimmel et al., 1995). As a result, the CES of 5% has been the most used default value for continuous data. To better align with biological relevance, a theory has been proposed to scale the CES expressed as a percent change to the maximum response (the general theory of effect size, GTES) (Slob, 2017). Suggestions for selecting endpoint specific CES values have also been made, advocating that historical data and expert judgement should be used on a case-by-case basis (Buist et al., 2009; Dekkers et al., 2006). However, in situations where multiple endpoints are analyzed, a unified CES is preferred (Vieira Silva et al., 2021).

The US EPA Benchmark Dose Technical Guidance recommends always reporting the BMD estimate with CES in terms of a difference in means equal to 1SD (US EPA, 2012a). In contrast to the US EPA, the EFSA Scientific Committee argues that the associated BMD depends on the particular study due to study-specific factors (measurement error; dosing error; heterogeneity in experimental conditions). Another challenge of using the 1SD metric is that the associated BMD estimate cannot be translated into an equipotent dose for populations with greater within-group variation. Therefore, EFSA recommends defining the CES as a percent change in the mean response relative to the background response, with 5% as the default option for continuous data (EFSA Scientific Committee, 2017).

The BMD modeling requires several critical decisions that must be addressed and transparently documented. Beyond selecting a CES, choosing an appropriate modeling approach is essential, as the outcome can vary significantly depending on the model used. For instance, non-sigmoid exponential models have been shown to produce falsely high BMDLs, potentially leading to underestimated risk (Ringblom et al., 2014). Additionally, Sand and colleagues demonstrated that model dependence of the BMDL estimate was more pronounced at lower levels of CES, indicating that the selection of an effect/response level is a critical decision that can significantly influence the outcome (Sand et al., 2002). Multimodel estimation and inference using model averaging is considered to be a reliable method to account for model uncertainty while also addressing the uncertainty related to sampling errors in the data (Wheeler and Bailer, 2007; Wheeler and Bailer, 2008a; Wheeler and Bailer, 2008b; Wheeler and Bailer, 2009). In model averaging, the individual model results are combined via model weighing, with higher weights for models that fit the data better. These weights are often defined in terms of the Akaike information criterion (AIC). Another aspect of CES selection is the suitability criteria for assessing the quality of a BMDL value.

In 2022, the EFSA Scientific Committee published updated guidance on the use of the BMD approach in risk assessment (EFSA Scientific Committee, 2022). The purpose of this update was to further support the implementation of dose-response modeling in EFSA’s work and to harmonize the theoretical insights between EFSA and other national and international organizations, such as WHO and US EPA (US EPA, 2012b; WHO, 2009; WHO, 2020). The US EPA, for example, offers criteria across models (Haber et al., 2018). Although model averaging addresses many differences, criteria for BMD confidence/credible interval width remain essential. Alternatives to BMDL are advised if the BMD is 10 times lower than the lowest non-zero dose or if the BMDU/BMDL ratio is over 50. The EFSA Scientific Committee in their guidance advises using the same criteria to determine if the width of the BMD confidence/credible interval should be considered by the risk assessor (EFSA Scientific Committee, 2022). In this most recent guidance on BMD modeling EFSA advises the use of Bayesian based analyses instead of the previously recommended frequentist approach (EFSA Scientific Committee, 2017; EFSA Scientific Committee, 2022). Unlike frequentist probability, which is based on the expected frequency of an event, Bayesian thinking conceives of probability as a measure of strength of belief. Bayesian analysis combines prior information (represented by a mathematical probability distribution, the prior) with data from the study (the likelihood function) to generate an updated probability distribution (the posterior) representing the information available for decision-making. The methodological differences between Bayesian and frequentist approaches for the analysis of medical research are discussed elsewhere (Goligher et al., 2024; Shao and Shapiro, 2018). In contrast to the EFSA Scientific Committee and Shao and Shapiro, Goligher and colleagues argue that Bayesian and frequentist methods should be viewed as complementary rather than as rivals. However, the impact of shifting from frequentist to Bayesian on risk assessment outcomes remains to be studied.

PFAS are abundant environmental pollutants–often highly mobile, persistent and bioaccumulative–posing significant regulatory challenges (OECD, 2021). EFSA’s assessments of PFAS have evolved from using the NOAEL approach (EFSA CONTAM Panel, 2008), to the BMD approach (EFSA CONTAM Panel, 2018; EFSA CONTAM Panel, 2020), resulting in a wide range of tolerable intake levels for PFOS–from 150 ng/kg b.w./day (EFSA CONTAM Panel, 2008), to 13 ng/kg b.w./week (EFSA CONTAM Panel, 2018), and further to 4.4 ng/kg b.w./week for the sum of four PFAS (EFSA CONTAM Panel, 2020). In their first assessment from 2008, EFSA based the PoD on altered serum concentrations of a thyroid hormone and on serum cholesterol levels in monkeys, using the NOAEL approach (EFSA CONTAM Panel, 2008; Seacat et al., 2002). Subsequent assessments in 2018 and 2020 used a frequentist BMD approach for a CES of 5% reduction of serum cholesterol in adults (EFSA CONTAM Panel, 2018; Steenland et al., 2009), and a CES of 10% reduction of antibody response in children (Abraham et al., 2020; EFSA CONTAM Panel, 2020), respectively. The US EPA applied a frequentist BMD approach and a 5% reduction of decreased antibody concentrations in children (Grandjean et al., 2012), to derive a reference dose of 0.0079 ng/kg b.w. for PFOS (US EPA, 2022a). Due to their high regulatory relevance, these four studies were selected as case studies for the present study. The overarching question is: How does the selection of a CES and the statistical approach influence the PoD for risk assessment from both quantitative and qualitative perspectives?

## 2 Materials and methods

### 2.1 Case studies – animal data

In the first case study (Seacat et al., 2002), male and female cynomolgus monkeys were administered the potassium salt of PFOS at 0 (n = 6/sex), 0.03 (n = 4/sex), 0.15 (n = 6/sex) or 0.75 (n = 6/sex) mg/kg b.w. per day via oral gavage for 26 weeks. Significant adverse toxicity was observed at the highest dose, which included mortality of two out of six male monkeys (the authors concluded the probable causes of death to be pulmonary inflammation in one case and hyperkalemia in the other), decreased body weights, increased liver weights with hepatocellular hypertrophy and vacuolization, lowered serum total cholesterol, increased levels of high-density lipoprotein (HDL) cholesterol, lowered total triiodothyronine (TT3) concentrations (without evidence of hypothyroidism), and lowered estradiol levels. In the middle dose groups, the following changes were observed: lowered levels of HDL (females), increased levels of TSH (males) and lowered TT3 concentrations (males and females). No adverse effects occurred at a dose of 0.03 mg/kg bw per day as compared to the control group. However, it is important to note that the number of animals per group was rather low thereby reducing the statistical power for groupwise comparisons. In their initial risk assessment of PFOS, the EFSA Scientific Committee identified reduced levels of TT3 and increased levels of HDL cholesterol at termination (day 82) as critical effects and used groupwise comparison to derive a NOAEL of 0.03 mg/kg b.w. per day (EFSA CONTAM Panel, 2008). In the present study, mean values and SD for TT3 and HDL from the most sensitive sex (females) was used for dose-response assessment.

### 2.2 Case studies – human data

The second case study (Steenland et al., 2009), investigated associations between PFOS and PFOA and serum lipids in a cross-sectional study within the C8 cohort, which included approximately 46,000 adults over 18 years of age who were not taking cholesterol-lowering medication. Median PFOS and PFOA levels were 20 and 27 ng/mL respectively. Although the magnitude of the association was modest (approximately a 4% increase in total cholesterol from the lowest deciles to the medians), the odds ratio (OR) for high cholesterol increased by 40%–50% from the lowest to the highest quartiles of PFOS and PFOA. The increase in total serum cholesterol was chosen as a critical endpoint both for PFOS and PFOA exposures in EFSA’s CONTAM panel’s scientific opinion (EFSA CONTAM Panel, 2018). For the present study, the mean and SD for deciles were used to assess the dose-response relationships.

In the third case study (Grandjean et al., 2012), prenatal (∼gestation week 32) and postnatal (children age 5 years) exposures to PFOS and PFOA and their association with offspring post-booster antibody concentrations to diphtheria at 5 and 7 years of age were examined. The study involved a strong interventional component where antibody production was initiated through vaccination, and the increase in antibody concentrations was followed prospectively in relation to baseline concentrations of PFOS and PFOA. This study was previously identified by the EFSA CONTAM Panel in 2018 to comprise a potential critical endpoint and was further used as the key study by the US EPA in 2022 (EFSA CONTAM Panel, 2018; US EPA, 2022a). In the present study, the mean and SD for deciles were used to assess the dose-response relationships.

The fourth case study (Abraham et al., 2020), examined a cohort of 101 infants from Germany to investigate the association between plasma concentrations of PFAS-4 (the sum of PFHxS, PFOS, PFOA, and PFNA) and antibodies to Diphtheria, Tetanus, and *Haemophilus* influenzae type b (Hib). PFOA concentrations in infant plasma were significantly and inversely correlated with antibody concentrations. Upon request, the authors provided a graph, from which individual data points were extracted using Graph Grabber (version 2.0.2, Quintessa Ltd.). The data points were independently extracted, and the results were compared by two researchers for quality control and to avoid miscalculations. Deciles were then derived and used for dose-response assessment.

### 2.3 Frequentist benchmark dose-response analysis

Frequentist benchmark dose modeling was performed in R (version 4.2.2; R Development Core Team, 2022), using the package PROAST (version 70.3), developed by the National Institute for Public Health and the Environment of the Netherlands (RIVM). The following model families were employed: Exponential, Hill, Inverse exponential, and Log-normal. Model selection was guided by the likelihood-ratio method for model fitting and the Akaike information criterion (AIC) for model comparison and selection. The AIC was set at a threshold of 2 for comparing and choosing the best-fitting models. The reported doses or concentrations were treated as independent variables and the observed effects as dependent variables.

To gain deeper insight into the dose-response data, model averaging (MA) was employed using a bootstrap method, with model weights determined by the AIC and 500 iterations (EFSA Scientific Committee, 2017; Moerbeek et al., 2004; Varewyck and Verbeke, 2019; Wheeler and Bailer, 2007). BMD values were extracted from individual bootstrap runs of the model averaging analyses, and the BMD medians were used for subsequent comparisons. The BMDLs presented, are the lower bounds of the 90% confidence intervals of the BMD estimates.

### 2.4 Bayesian benchmark dose-response analysis

Bayesian-based benchmark dose modeling was conducted using EFSA’s Bayesian BMD web application, based on R (version 4.3.2, 2023-10-31) using the BMABMDR R-package (Kremer et al., 2022). Only continuous summary data from the respective treatment groups for the animal case study, and from deciles for the epidemiological case studies, were used for analysis.

To standardize prior distribution specification and ensure comparability across all modeled case studies, the non-informative default PERT distribution was applied. The detailed mathematical background and its application are described elsewhere (EFSA Scientific Committee, 2022; Kremer et al., 2022). In brief, the default prior selection was used, with natural and technical model parameters set by EFSA’s Bayesian BMD platform for an extended dose range. Bridge sampling was applied with 30,000 draws from the posterior distribution. The number of Monte Carlo Markov Chains (MCMCs) was set to 3, with 3,000 iterations, and 1,000 MCMC iterations discarded as a warmup. Models were selected from default model families for normal and lognormal distributions. Estimates for BMDL, BMD and BMDU were derived using weighted model averaging as described above.

A non-default approach was necessary in one case-study (Steenland et al., 2009), as EFSA’s Bayesian BMD platform did not provide a meaningful background estimate for the original dataset using the default method. In this instance, the priors were set manually: the central estimate of the lowest decile +/− 1SD was used as the background prior, and the central estimate of the highest decile +/− 1SD was used as the prior for the maximum response level.

Prior settings for the BMD estimates – “most likely,” “min” and “max” – were left unchanged, as derived from EFSA’s Bayesian BMD platform for the remaining case studies. Detailed modeling settings of all case studies are available in the Supplementary Table S1.

### 2.5 Selection of critical effect size (CES)

The selection of the CES involved testing four methods: the 5% and 10% default approaches (EFSA Scientific Committee, 2017), the 1SD approach (US-EPA, 2012a) and, where applicable, an effect size defined by the General Theory of Effect Size (GTES) (namely, $\sqrt[{8}]{M}$ , where M is the maximum response) (Slob, 2017). The CES values based on 1SD were derived from the standard deviations of the group means for the respective lowest deciles, while CES values based on GTES were derived via frequentist-based modeling and applied in both frequentist and Bayesian BMD analyses.

In the frequentist BMD modeling process, for case-study 1 (Seacat et al., 2002), the complete dataset was used, with the more susceptible group of female monkeys selected through the PROAST script. The respective endpoints HDL or TT3 were modeled separately for each CES. The remaining case studies did not have datasets with defined sex strata, so pooled quintiles for both sexes were used as derived from the original studies. For case-study 2 (Steenland et al., 2009), datasets on PFOA and PFOS exposure with endpoint total cholesterol (TC), were modeled separately for each CES. For case study 3 (Grandjean et al., 2012), datasets on PFOA and PFOS exposure with endpoint anti-diphtheria toxoid antibody serum titer, were similarly analyzed with different CES values. In case study 4 (Abraham et al., 2020), the dataset on summed exposure to PFOA, PFOS, PFHxS and PFNA was modeled using the anti-diphtheria toxoid antibody serum titer endpoint, again with the different CES values.

## 3 Results

The results are presented individually for each case, with the overarching findings and conclusions summarized in the discussion section.

### 3.1 Case study 1

The two endpoints with the lowest observed effect levels were selected for further investigation. These endpoints include increased HDL and decreased TT3 levels in female monkeys at termination (Seacat et al., 2002).

Table 1 presents the BMD modeling results using various CES selection options. Frequentist modeling for HDL yielded BMDL estimates ranging from 61 to 6,710 ng/mL, with central BMD estimates between 5,702 and 46,455 ng/mL. Higher values were calculated for the reduction in serum TT3, with BMDLs ranging from 771 to 13,500 ng/mL, and central BMDs from 10,070 to 45,535 ng/mL.

**TABLE 1:** Case study 1. Benchmark Dose (BMD) modeling of serum high-density lipoprotein (HDL) cholesterol and total triiodothyronine (TT3) in cynomolgus monkeys exposed orally to PFOS (Seacat et al., 2002), using frequentist and Bayesian approaches across four Critical Effect Size (CES) options.

	Frequentist				Bayesian			
CES (%)	BMDL (ng/mL)	BMD (ng/mL)	BMDU (ng/mL)	BMDU/BMDL	BMDL (ng/mL)	BMD (ng/mL)	BMDU (ng/mL)	BMDU/BMDL
High-density lipoprotein (HDL)								
5	61	5,702	33,800	550	5,802	28,620	1.0E+5	17
10	373	12,420	50,700	136	9,724	33,391	1.0E+5	10
28.6 (1SD)	6,710	46,455	1.0E+5	15	26,368	63,597	1.2E+5	4.7
25.6 (GTES)	4,030	34,920	92,500	23	22,703	58,391	1.2E+5	5.1
Total triiodothyronine (TT3)								
5	771	10,070	37,500	49	7,837	24,446	65,579	8.4
10	3,610	22,280	56,400	16	14,278	37,661	78,173	5.5
17.9 (1SD)	13,500	45,535	86,300	6.4	28,775	60,304	1.0E+5	3.6
13 (GTES)	5,230	26,740	67,000	13	19,132	46,273	88,003	4.6

BMD, benchmark dose; BMDL, lower bound BMD; BMDU, upper bound BMD; CES, critical effect size; SD, standard deviation; GTES, general theory of the effect size.

For Bayesian modeling, BMDLs for HDL were estimated between 5,802 and 26,368 ng/mL, with central BMDs ranging from 28,620 to 63,597 ng/mL. Similarly, Bayesian modeling for TT3 data resulted in higher values compared to the frequentist approach, with BMDLs ranging from 7,837 to 28,775 ng/mL and central BMDs from 24,446 to 60,304 ng/mL.

As expected, BMD values increased with higher CES levels. However, the BMDU/BMDL ratio calculated using the frequentist approach exceeded 100 when the CES was set at 5% or 10% for HDL. Only in the case of a CES of 17.9% (equivalent to 1SD reduction) for TT3 was the BMDU/BMDL ratio below 10. In contrast, Bayesian modeling generally produced BMDU/BMDL ratios below 10 across all CES options for both endpoints, except for a 5% CES for HDL, which yielded a ratio of 17.

**FIGURE 1:**
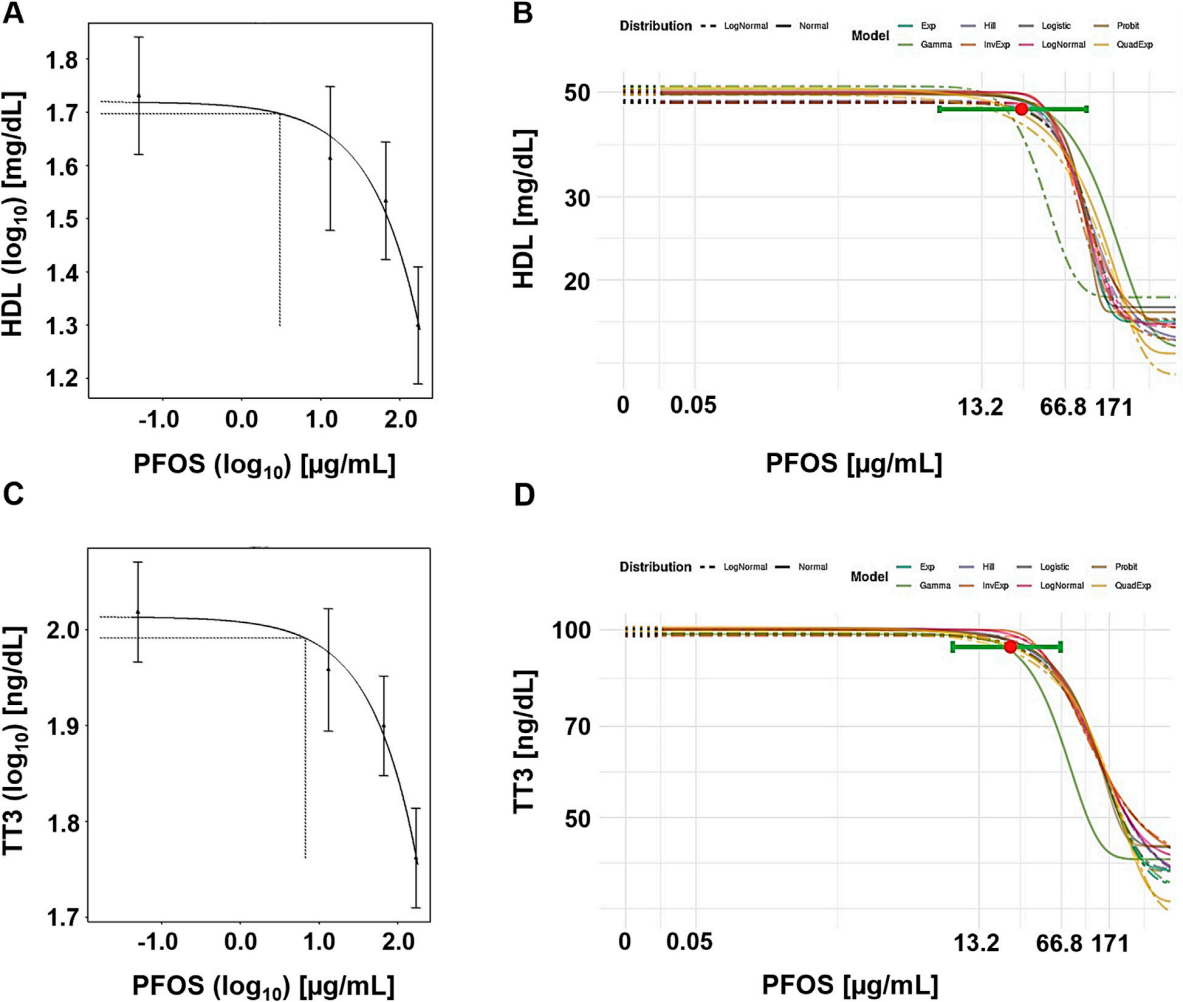
Case study 1. Example plots of Benchmark Dose (BMD) modeling of serum high-density lipoprotein (HDL) cholesterol **(A, B)** and total triiodothyronine (TT3) **(C, D)** in cynomolgus monkeys exposed orally to PFOS (Seacat et al., 2002), using frequentist and Bayesian approaches and a 5% change as the critical effect size (CES). **(A)** Frequentist approach using the exponential model m3, with dotted lines indicating a 5% change under parameter a, and the corresponding central BMD estimate (double-logarithmic scale). **(B)** Bayesian approach showing all fitted models, with model averaged central BMD estimate (red dot) and credible interval (horizontal green bar). **(C)** Frequentist approach using the exponential model m3, with dotted lines indicating a 5% change under parameter a, and the corresponding central BMD estimate (double-logarithmic scale). **(D)** Bayesian approach showing all fitted models, with model averaged central BMD estimate (red dot) and credible interval (horizontal green bar).

Figure 1 displays example plots for the lowest option, a CES of 5%. Notably, the central estimate (BMD) in the frequentist approach is located below the lowest tested dose, while the Bayesian approach places the central BMD between the low and middle experimental doses.

### 3.2 Case study 2

In this case study, data on total cholesterol changes in a human population exposed to PFOA and PFOS (Steenland et al., 2009) were analyzed.

The BMD-modeling results using various CES selection options were notably influenced by the large variation in serum cholesterol levels among human subjects. Central BMD values calculated for PFOA using the frequentist approach ranged from 0.004 ng/mL to 136 ng/mL (Table 2). The very wide confidence intervals, with BMDU/BMDL ratios ranging from 524 to 5.1E+5 indicate the difficulty in achieving a precise estimate. Using the Bayesian approach, it was not possible to establish a BMD value for CESs above 5%, as they significantly exceeded the maximum observed response level. For a CES of 5%, the BMD was estimated at 15 ng/mL, with a credible interval ranging from 2.1 to 34 ng/mL.

**TABLE 2:** Case study 2. Benchmark Dose (BMD) modeling of total serum cholesterol in a human cohort exposed to PFOA and PFOS via contaminated drinking water (Steenland et al., 2009), using frequentist and Bayesian approaches across four Critical Effect Size (CES) options.

	Frequentist				Bayesian			
CES (%)	BMDL (ng/mL)	BMD (ng/mL)	BMDU (ng/mL)	BMDU/BMDL	BMDL (ng/mL)	BMD (ng/mL)	BMDU (ng/mL)	BMDU/BMDL
PFOA								
5	4.6E-5	0.004	0.024	524	2.1	15	34	17
10	5.6E-5	0.043	0.473	8,462	N/A	N/A	N/A	N/A
30.2 (1SD)	0.007	90	3,580	5.1E+5	N/A	N/A	N/A	N/A
33.1 (GTES)	0.022	136	1,700	78,704	N/A	N/A	N/A	N/A
PFOS								
5	8.6E-6	0.002	7.8	9.1E+5	9.6	19	27	2.8
10	4.1E-4	0.081	31	76,790	19	51	116	6.2
31.5 (1SD)	0.806	24	363	450	N/A	N/A	N/A	N/A
35.0 (GTES)	2.0	86	508	258	N/A	N/A	N/A	N/A

BMD, benchmark dose; BMDL, lower bound BMD; BMDU, upper bound BMD; CES, critical effect size; SD, standard deviation; GTES, general theory of the effect size; N/A, not available.

**FIGURE 2:**
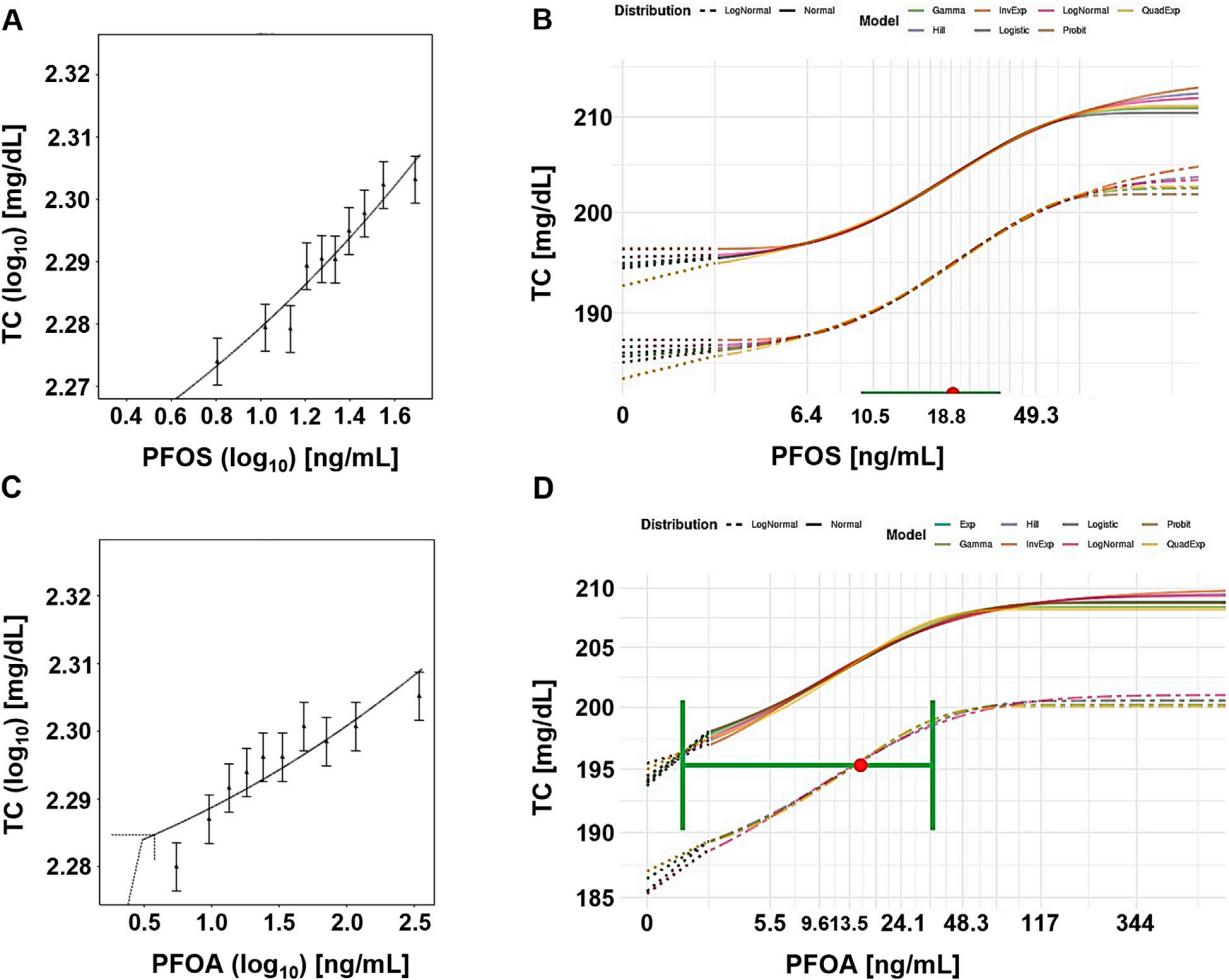
Case study 2. Example plots of Benchmark Dose (BMD) modeling of total serum cholesterol (TC) in a human cohort exposed to PFOS **(A, B)** and PFOA **(C, D)**, via contaminated drinking water (Steenland et al., 2009), using frequentist and Bayesian approaches and a 5% change as the critical effect size (CES). **(A)** PFOS, frequentist approach using the exponential model m3 (double-logarithmic scale). **(B)** PFOS, Bayesian approach, showing all fitted models, with model averaged central BMD estimate (red dot) with credible interval (horizontal green bar). **(C)** PFOA, frequentist approach using the exponential model m5, with dotted lines indicating a 5% change over parameter a, and the corresponding central BMD estimate (double-logarithmic scale). **(D)** PFOA, Bayesian approach showing all fitted models, with model averaged central BMD estimate (red dot) and credible interval (horizontal green bar).

For PFOS, the central BMD estimates from the frequentist approach ranged from 0.002 ng/mL to 86 ng/mL (Table 2). The BMDU/BMDL ratios ranged from 258 to 9.1E+5. Although these ratios are lower compared to the ratios for PFOA, they still indicate significant uncertainty. The Bayesian approach was unable to establish any values at CES above 10%. However, the central BMD estimates at CES of 5% and 10% were 19 ng/mL (BMDU/BMDL = 2.8) and 51 ng/mL (BMDU/BMDL = 6.2), respectively. Figure 2 presents example plots from the frequentist approach (single exponential model) and the Bayesian approach (all models included for model averaging).

### 3.3 Case study 3

Case study 3 involves a study in children that found reduced antibody serum titers associated with PFAS blood concentrations (Grandjean et al., 2012).

Table 3 presents the BMD modeling results using various CES selection options. The central BMD estimates for PFOA from the frequentist mode ranged from 1.8 to 6.9 ng/mL. The tight clustering of these estimates indicates a steep dose-response curve at the high end of exposure. Significant interindividual variation within the population is reflected in the inability to establish BMD values based on the 1SD CES (135% of the mean of the lowest decile). Additionally, the BMDU/BMDL ratios were generally very high (>100,000) for the frequentist approach.

**TABLE 3:** Case study 3. Benchmark Dose (BMD) modeling of anti-diphtheria toxoid antibody serum titer in a cohort of Faroe children, exposed to PFOA and PFOS (Grandjean et al., 2012), using frequentist and Bayesian approaches across four Critical Effect Size (CES) options.

	Frequentist				Bayesian			
CES (%)	BMDL (ng/mL)	BMD (ng/mL)	BMDU (ng/mL)	BMDU/BMDL	BMDL (ng/mL)	BMD (ng/mL)	BMDU (ng/mL)	BMDU/BMDL
PFOA								
5	2.2E-6	1.8	3.9	1.8E+6	0.197	1.1	2.3	11
10	3.5E-6	2.4	4.5	1.3E+6	0.337	1.2	2.4	7.1
135 (1SD)	N/A	N/A	N/A	N/A	N/A	N/A	N/A	N/A
73 (GTES)	8.1E-5	6.9	8.5	1.1E+5	3.4	6.1	12	3.5
PFOS								
5	1.3E-6	0.046	11	9.0E+6	1.7	6.6	12	7.1
10	1.7E-6	0.114	14	7.9E+6	2.6	7.7	13	5.0
68.4 (1SD)	N/A	N/A	N/A	N/A	N/A	N/A	N/A	N/A
58.3 (GTES)	1.3E-5	1.4	33	2.5E+6	14	23	43	3.1

BMD, benchmark dose; BMDL, lower bound BMD; BMDU, upper bound BMD; CES, critical effect size; SD, standard deviation; GTES, general theory of the effect size; N/A, not available.

The central BMD estimates for PFOA from the Bayesian approach ranged from 1.1 to 6.1 ng/mL. In contrast to the extreme confidence intervals from the frequentist approach, the credible intervals for these Bayesian estimates were narrow, with BMDU/BMDL ratios ranging from 3.5 to 11. Notably, the BMD estimates from both approaches returned similar values, but vastly different measures of uncertainty. The large interindividual variation in the population is also reflected in the inability to establish BMD values based on the 1SD CES.

For PFOS, the central BMD estimates from the frequentist approach showed a strong dependence on the CES selection (Table 3). The BMD values ranged from 0.046 ng/mL to 1.4 ng/mL with very broad confidence intervals (>10,000). In comparison, the central BMD estimates from the Bayesian model were 1–2 orders of magnitude higher, ranging from 6.6 ng/mL to 23 ng/mL. However, the credible intervals produced BMDU/BMDL ratios in the range of 3.1–7.1.

**FIGURE 3:**
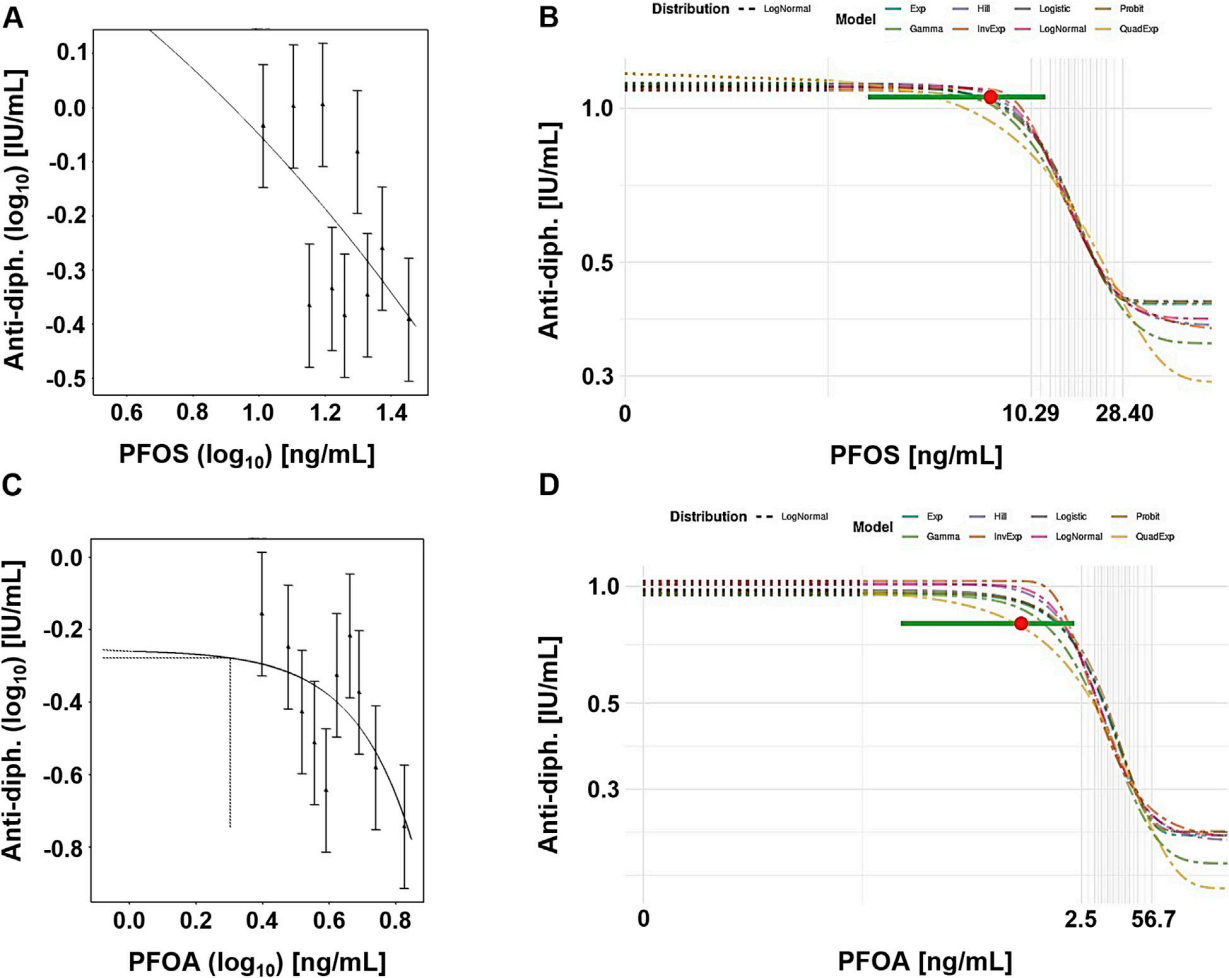
Case study 3. Example plots of Benchmark Dose (BMD) modeling of anti-diphteria toxoid antibody serum titer (anti-diph.) and PFOS **(A, B)** and PFOA **(C, D)** levels, in a cohort of children (Grandjean et al., 2012), using frequentist and Bayesian approaches and a 5% change as the critical effect size (CES). **(A)** PFOS, frequentist approach using the exponential model m3 on a double-logarithmic scale. **(B)** PFOS, Bayesian approach showing all fitted models, with model averaged central BMD estimate (red dot) with credible interval (horizontal green bar), **(C)** PFOA, frequentist approach using the exponential model m3, with dotted lines indicating a 5% change under parameter a, and the corresponding central BMD estimate (double-logarithmic scale). **(D)** PFOA, Bayesian approach showing all fitted models, with model averaged central BMD estimate (red dot) with credible interval (horizontal green bar).

Figure 3 shows example plots from the frequentist (single exponential model) and Bayesian (model averaging) approaches. In both cases, the central BMD for a CES of 5% was calculated at a dose below the lowest decile.

### 3.4 Case study 4

The final case study focused on epidemiological data from children, specifically examining their antibody serum titers for diphteria and PFAS blood concentrations (Abraham et al., 2020). The exposure was measured as the sum of four PFAS (PFOS, PFOA, PFHxS and PFNA).

The frequentist BMD-modeling results using various CES selection options produced central BMD estimates ranging from 31 ng/mL to 53 ng/mL (Table 4). As expected, higher CES resulted in higher BMD estimates. The BMDU/BMDL ratios ranged from 1.5 to 9.4. The Bayesian approach showed slightly lower values, with central BMD estimates of 29 ng/mL and 32 ng/mL, for CES values of 5% and 10%, respectively. When the GTES was used to define the CES (54.3%), the Bayesian approach yielded a slightly higher BMD value (63 ng/mL) compared to the frequentist approach (53 ng/mL). The credible intervals closely resembled the confidence interval calculated using the frequentist approach (from 4.1 to 15).

**TABLE 4:** Case study 4. Benchmark Dose (BMD) modeling of anti-diphteria toxoid antibody serum titer in a cohort of German children, exposed to PFAS-4 (PFOS, PFOA, PFHxS, PFNA) (Abraham et al., 2020), using frequentist and Bayesian approaches across four Critical Effect Size (CES) options.

	Frequentist				Bayesian			
CES (%)	BMDL (ng/mL)	BMD (ng/mL)	BMDU (ng/mL)	BMDU/BMDL	BMDL (ng/mL)	BMD (ng/mL)	BMDU (ng/mL)	BMDU/BMDL
PFAS-4 (PFOS, PFOA, PFHxS, PFNA)								
5	4.2	31	40	9.4	3.9	29	58	15
10	7.7	36	44	5.7	5.6	32	54	10
121 (1SD)	N/A	N/A	N/A	N/A	N/A	N/A	N/A	N/A
54.3 (GTES)	38	53	57	1.5	34	63	138	4.1

BMD, benchmark dose; BMDL, lower bound BMD; BMDU, upper bound BMD; CES, critical effect size; SD, standard deviation; GTES, general theory of the effect size.

**FIGURE 4:**
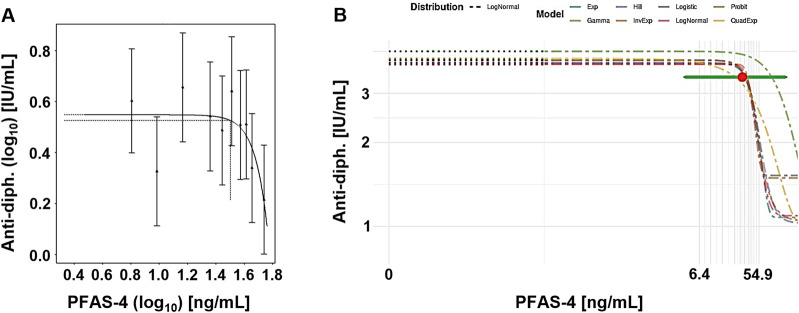
Case study 4. Example plots of Benchmark Dose (BMD) modeling of anti-diphteria toxoid antibody serum titer (anti-diph.) and the summed concentration of PFAS-4 (PFOS, PFOA, PFHxS and PFNA), in a cohort of German children (Abraham et al., 2020), using frequentist and Bayesian approaches at a CES 5%. **(A)** Frequentist approach using the exponential model m3, with dotted lines indicating a 5% change under parameter a, and the corresponding central BMD (double-logarithmic scale). **(B)** Bayesian approach showing all fitted models, model-averaged central BMD estimate (red dot) with credible interval (horizontal green bar).

Figure 4 presents example plots from the frequentist approach (single exponential model) and Bayesian approach (model average). Although there is a large variation in the data, the dose response curve indicates a steep response at the higher end of exposure. However, the confidence/credible intervals do cover the entire range of exposure.

## 4 Discussion

The literature in regulatory toxicology underscores a notable divergence in guidance values stemming from methodological differences between the NOAEL and BMD approaches. Several studies highlight the limitations of the NOAEL approach, which often neglects valuable dose-response information (Bokkers and Slob, 2005; Haber et al., 2018). In contrast, the BMD method, recommended by agencies like the EFSA and the US EPA, is regarded as more robust, providing a precise PoD by utilizing the entire dose-response curve and accounting for uncertainty (EFSA Scientific Committee, 2022; US EPA, 2012b). Nevertheless, even within the BMD framework, choices regarding the CES and statistical model selection can lead to significantly different outcomes (Ringblom et al., 2014; Sand et al., 2002). This study, therefore, aims to analyze CES selection in risk assessment, with a particular emphasis on regulatory evaluations of PFAS. How does the CES and statistical approach for dose-response assessment impact the PoD required for deriving guidance values? In this study, a comparative analysis of four key studies collected from recent risk assessment reports was undertaken. Both the frequentist and the Bayesian multimodel inference techniques were explored to also assess how varying statistical frameworks influence outcomes in terms of BMD values and their level of certainty and to account for paradigm shifts in current international guidance for BMD modeling.

### 4.1 Case study 1

In the first case study, cynomolgus monkeys were exposed to varying doses of PFOS (Seacat et al., 2002). The study identified a NOAEL at 0.15 mg/kg b.w./day, but EFSA’s CONTAM panel deemed the altered serum levels of TT3 and HDL at this dose as adverse, pushing their derived NOAEL down to 0.03 mg/kg b.w./day (the lowest dose tested) (EFSA CONTAM Panel, 2008).

Our re-evaluation using BMD modeling, based on PFOS serum concentration rather than administered dose, shows that BMDL values varied considerably (from 61 to 28,775 ng/mL) depending on the endpoint, the choice of modeling framework and the CES (Table 1). Two general trends observed in this case were firstly, that BMD estimates increased as larger CES values were applied, and secondly, that the Bayesian models producing consistently narrower BMDU/BMDL ratios.

Using a CES of 5%, the frequentist BMDL_05_ for HDL (61 ng/mL) was far below EFSA panel’s NOAEL serum concentration (13,200 ng/mL), which raises concerns about overly conservative BMDL estimates. In contrast, the Bayesian BMDL_05_ (5,802 ng/mL) aligned more closely with EFSA’s reference point. Another observation is that higher CES values (e.g., 1SD and GTES) might result in more biologically relevant reduction levels. In this first case study, the 1SD and GTES options for CES were close to the lower “normal” range.

For example, the study by Seacat and colleagues shows a background level of HDL in serum of about 50 mg/dL,HDL normally ranges from 30 mg/dL to 150 mg/dL according to Seacat et al. (2002). A CES of 5% or 10% would according to our models result in a reduction of serum HDL to 49.9 mg/dL and 47.2 mg/dL, respectively–i.e., very close to background and well within the range of serum HDL considered as normal for the test species. The 1SD as well as the GTES describe response levels closer to the lowest cut-off of 30 mg/dL and more importantly return BMD estimates with narrow confidence/credible intervals.

While the 5% CES is widely used in regulatory settings (EFSA Scientific Committee, 2017), our results indicate it may lead to excessive conservatism in cases with high variability, potentially undermining the robustness of conclusions. In contrast, higher CES values produced more reliable estimates with reduced intervals, supporting their application in such contexts. From a regulatory perspective, the choice of CES must balance conservatism with biological relevance. A 10% CES, analyzed with Bayesian statistics, may offer a promising compromise. For example, the Bayesian BMDL_10_ for HDL was 9,724 ng/mL, with a more constrained BMDU/BMDL ratio as compared with the frequentist BMDL_10_. However, it is important to recognize that a 10% reduction in HDL is not equivalent to a NOAEL but can be considered more comparable to the lowest observed adverse effect level (LOAEL). This may therefore have an impact on the need for assessment factors. For example, the ECHA guideline (chapter R.8; characterization of dose-response for human health) suggests using an assessment factor of 3 as minimum if the PoD is a LOAEL rather than a NOAEL.

What difference would a change from NOAEL to BMD lead to in the derivation of a tolerable intake value? To answer this hypothetical question, we approximated administered doses using a second order polynomial equation to describe the relationship between the internal concentrations and the administered doses (see Supplementary Figure S1). The frequentist and Bayesian BMDL_05_ values were then attributed to approximately 0.0068 and 0.012 mg/kg b.w. per day, respectively. With the same total assessment factor of 200 used by EFSA (EFSA CONTAM Panel, 2008), this would result in a TDI of 34 ng/kg b.w./day via the frequentist approach and 60 ng/kg b.w./day via the Bayesian approach.

If we instead base the calculation on the BMDL_10_ and treat this CES as a LOAEL, an additional assessment factor of 3 would be needed to extrapolate to a no-effect level. This adjustment increases the total assessment factor of 600 (x100 for inter- and intraspecies variation, x2 for subchronic to chronic, and x3 for extrapolation to a no-effect level), yielding a TDI of 35 ng/kg b.w./day via the frequentist approach and 82 ng/kg b.w./day via the Bayesian approach, as opposed to the TDI of 150 ng/kg b.w./day, established by EFSA in 2008 (EFSA CONTAM Panel, 2008). These discrepancies highlight the need for a transparent and balance approach to risk assessment, one that carefully weighs conservatism against biological relevance when selecting CES values and statistical frameworks.

### 4.2 Case study 2

The second case study examined the associations between PFOS/PFOA exposure and serum lipid levels within a large cohort of from the state of West Virginia (the C8 cohort) (Steenland et al., 2009). This cross-sectional study found modest associations between serum levels of PFOS/PFOA and increased total cholesterol, with odds ratios for high cholesterol rising notably from the lowest to highest exposure quartiles. The study’s findings played a critical role in informing the EFSA CONTAM Panel’s opinion, as the increase in total serum cholesterol was identified as a key endpoint in the 2018 report (EFSA CONTAM Panel, 2018).

Our BMD modeling revealed significant uncertainties due to the large variation in serum cholesterol levels among subjects (Table 2). The frequentist approach produced wide-ranging BMD values particularly at higher CES values, indicating difficulty in establishing precise estimates. Similarly, the Bayesian approach struggled to establish a reliable BMD for CES above 5%, reflecting the limitations of the method at effect sizes outside the observed range. However, at a CES of 5%, the Bayesian approach produced more stable results, with narrower credible intervals compared to the frequentist approach.

The biological relevance of increased total cholesterol needs to be addressed in the selection of CES. Even a small (5%) increase in cholesterol has been associated with higher risk for cardiovascular disease (EFSA CONTAM Panel, 2018; Lewington et al., 2007; Mihaylova et al., 2012; Piepoli et al., 2016). However, the results from our study highlight the challenge of deriving reliable BMD estimates in human populations, especially due to the high variability in endpoints like cholesterol levels and the relatively modest strength of associations. While Bayesian and frequentist methods provided different insights into dose-response relationships, both approaches underscored the significant uncertainty. The inability to establish CES values above 5% using Bayesian modeling suggests that the effects of PFOS and PFOA on cholesterol may not scale linearly at higher exposure levels.

The dose-response modeling of the C8 cohort data in EFSA’s 2018 report was heavily debated and contributed to its revision in 2020. The EFSA panel derived a BMDL_05_ of 9.4 ng/mL for PFOA using a single model and assumptions, including a fixed background level of 1 ng/mL. These choices resulted in a less steep dose-response curve and higher BMDL estimates compared to our study, where Bayesian modeling produced lower BMDL_05_ values. This discrepancy highlights the influence of methodological differences, particularly the use of single *versus* model-averaging approaches and assumptions about background exposure levels.

As a response to the 2018 EFSA report, both the European Chemicals Agency (ECHA) and the Dutch Institute for Health and Environment (RIVM) raised concerns about the robustness of the cholesterol data, noting the uncertainties in the dose-response (EFSA BIOCONTAM Panel, 2018). In specific, they pointed out that the reported 5% increase above the lowest decile is minimal, especially since cholesterol responses in rodents have been shown to level off at around 150%, suggesting the 5% change may not reflect a true maximum response for cholesterol, thereby referring to the GTES (Slob, 2017). This criticism seems to be in accordance with our observation, that no BMD was derived at higher CES. For continuous outcomes like cholesterol levels, the CES is usually defined as a percentage change from the background response (as calculated during the dose-response modeling). However, the CONTAM Panel redefined the CES as an increase relative to the lowest quantile, which makes the BMD calculation highly sensitive to the number of quantiles–using fewer quantiles raises the cholesterol level in the lowest group, leading to a higher BMD estimate.

For PFOA, the panel derived a TWI of 6 ng/kg b.w./week via PBPK modeling from their estimated BMDL_05_ of 9.4 ng/mL. For PFOS the study-specific TWI was calculated to 14 ng/kg b.w./week departing from a BMDL_05_ of 25 ng/mL. The final TWI for PFOS was later set to 13 ng/kg b.w./week, a median of three epidemiological studies. It is noticeable that the BMDL_05_ values calculated with Bayesian statistics in our study are significantly lower (2.1 ng/mL and 9.6 ng/mL, for PFOA and PFOS, respectively) compared with the PoD used by EFSA.

### 4.3 Case study 3

By examining antibody concentrations in children post-vaccination the third case study did not only track immune system performance over time but also highlighted the potential immunosuppressive effects of these chemicals (Grandjean et al., 2012). This study was recognized as a key study both by the EFSA, and the US EPA (EFSA CONTAM Panel, 2018; EFSA CONTAM Panel, 2020; US EPA, 2022a).

BMD modeling of the data showed distinct patterns for PFOA and PFOS (Table 3). For PFOA, the frequentist approach suggested a steep dose-response curve but exhibited significant uncertainty, with extremely wide BMDU/BMDL ratios, marking the results as unreliable. This uncertainty reflects a substantial interindividual variation, further evidenced by the inability to derive BMD values based on the CES from 1SD. In contrast, the Bayesian approach offered more stable estimates, demonstrating its potential for better handling of interindividual variability. For PFOS, frequentist BMD estimates varied greatly depending on CES selection and were accompanied by very broad confidence intervals, again highlighting unacceptable uncertainty. The Bayesian model produced more consistent and interpretable BMDL estimates, with narrower intervals compared to the frequentist approach.

Overall, the Bayesian approach offered more consistent and interpretable outcomes for both PFOA and PFOS, with significantly reduced BMDU/BMDL ratios compared to the frequentist method. PFOA appears more potent than PFOS, although both chemicals exhibit relatively steep dose-response curves. The biological relevance of a 5% reduction of vaccine titers should be carefully considered if this endpoint is selected as critical and as a base for CES selection. Following diphtheria vaccination, antibody titer typically exceeds 0.1 IU/mL, widely accepted as the protective threshold for immunity (WHO, 2017). Post-vaccination titers ideally range higher, which aligns with benchmarks for robust immunity. The implications of reduced titers for immune system development and long-term immunogenic impacts should not be overlooked.

The EFSA applied BMD modeling only for PFOS data from the study of Grandjean and colleagues, due to potential confounding with PFOA (EFSA CONTAM Panel, 2018; Grandjean et al., 2012). Using a frequentist approach and a single logarithmic model, they established a BMDL_05_ of 10.5 ng/mL for PFOS, with the first decile point of PFOS concentration as the reference background. However, this modeling approach was criticized during the following expert meeting (EFSA BIOCONTAM Panel, 2018), for using the lowest decile of antibody titers as a reference instead of extrapolating to a zero PFOS concentration. In their 2020 risk assessment, the Panel obtained additional data on the combined concentrations of combined PFAS exposure (PFAS-4) but ultimately identified a NOAEC of 27.0 ng/mL due to wide BMDU/BMDL intervals (EFSA CONTAM Panel, 2020). The US EPA also used the Grandjean study to derive a reference dose (7.9 × 10^−3^ ng/kg bw/day) (US EPA, 2022a). They directly refer to the BMDs for decreased antibody response to vaccines, published by Budtz-Jorgensen and Grandjean (Budtz-Jorgensen and Grandjean, 2018), as PoD for the final PFOA and PFOS reference doses. In that study the default CES of 5% was used and a regression model with log-transformed response variable was applied. By assuming no effect below the lowest observed concentration, the authors referred to “the most optimistic curve for extrapolating to zero exposure”. In their conclusion, the authors approximated a BMDL of 1 ng/mL serum for both PFOS and PFOA as appropriate (Budtz-Jorgensen and Grandjean, 2018). From our perspective, the US EPA could have provided a stronger and more transparent justification for the choice of CES, as was also commented by the Scientific Advisory Board of the US EPA (US EPA, 2022b).

Notably, the Bayesian BMDL_10_ values for PFOA and PFOS in our study fall within the same range as the BMDL_05_ 1 ng/mL used by the US EPA and about an order of magnitude lower than the BMDL_10_ by EFSA. This underscores the importance of transparency in CES selection supports the use Bayesian approaches for modeling epidemiological data with high variability.

### 4.4 Case study 4

In our last cast study, data on combined PFAS-4 exposure from 104 children were modeled using the anti-diphtheria toxoid antibody serum titer as the critical effect (Abraham et al., 2020). Both frequentist and Bayesian models produced BMDL estimates within a similar range (3.9 – 38 ng/mL) (Table 4). Each approach also yielded comparable BMDL values across tested CES levels, with relatively narrow confidence/credible intervals, enhancing confidence in these estimates. This case study uniquely reports that Bayesian statistics produced slightly lower BMDL values and slightly higher BMDU/BMDL ratios, when compared to the frequentist approach.

As with case study 3, substantial interindividual variation prevented the derivation of BMD values based on the 1SD CES, while the GTES give a CES of a significant (54%) reduction. The biological relevance of a CES at 5% or 10% remains debatable but the same arguments as for case study 3 apply here as well–the adversity of this endpoint relates to its potential impact on immune system development.

In 2022, the EFSA CONTAM Panel applied a CES of a 10% reduction in anti-diphtheria toxoid antibody serum titers, deriving a BMDL_10_ of 17.5 ng/mL (EFSA CONTAM Panel, 2020). In contrast, our study identified BMDL_10_ values of 7.7 ng/mL using the frequentist approach and 5.6 ng/mL Bayesian approach. This discrepancy appears to result from deviations in the modeling approaches. EFSA used the BMD as the PoD rather than the BMDL and applied a single model fit instead of model averaging via bootstrapping. EFSA argued, that model averaging produced unrealistically low BMDL values, below the BMDL values from individual models. Additionally, they noted that some curves from their bootstrap runs failed to level off at lower PFAS levels, which artificially lowered the BMDL estimates (EFSA CONTAM Panel, 2020).

Our reanalysis of the same data showed that the lowest measured PFAS-4 exposure was 2.5 ng/mL, which is lower than our BMDL_10_ values derived through model averaging. We observed a few bootstrap models that did not level off, but these cases should not pose major issues as long as the number of bootstrap runs is sufficiently large. To estimate a daily intake, the EFSA referenced a PBPK model to translate the internal PoD to a TWI of 4.4 ng/mg b.w./week (EFSA CONTAM Panel, 2020). Since our BMDL_10_ estimates are approximately half of their PoD, a corresponding TWI would likely be about 50% lower.

### 4.5 On the selection of CES

The selection of CES is a critical factor that significantly impacts BMDL estimates across all case studies. Generally, as CES values increase, BMD estimates rise, reflecting a greater degree of biological effect. Thus, the choice of CES is closely tied to the challenge of defining adverse from non-adverse effects. CES values of 5% or 10% are commonly recommended as protective thresholds, but higher CES values, like through the GTES, may yield BMDL estimates that are more biologically relevant. A BMDL based on a GTES-aligned CES represents a threshold close to, but not yet reaching, adverse effects. The balance between protective or relevant CES can be seen in immunotoxicity studies (such as Case Studies 3 and 4), where a 5% reduction in vaccine titers may not pose immediate health risks but could suggest early immune system perturbations, potentially leading to harm with increased exposure or in vulnerable populations. The GETS of approximately 50% may on the other hand indicate where to find a more direct threshold of adversity.

EFSA’s recent guidance on BMD modeling, moves away from default CES values and advocates for biologically justified CES values, with the proposal of creating a database of CES for common endpoints and species (EFSA Scientific Committee, 2022). Such an initiative could facilitate not only more informed CES selection but also provide a foundation for Bayesian modeling priors.

Our findings further indicate that higher CES values generally produce more reliable estimates, as shown by narrower confidence or credible intervals. This pattern is particularly evident in cases with high within-group variability, such as the lipid and immunological endpoints in Case Studies 1 and 3. Across most cases, the Bayesian approach yields narrower credible intervals. When an informative prior aligns well with the data, the Bayesian credible interval is often narrower than the frequentist confidence interval. However, if the prior conflicts with the data (e.g., the prior’s center significantly deviates from data trends), the resulting Bayesian credible interval may be broader than the frequentist confidence interval. This appears to be the case in Case Study 4, where the BMDU/BMDL ratio was larger for Bayesian BMD estimates, regardless of the CES.

Overall, the selection of a suitable CES ultimately balances conservatism, biological relevance, and precision based on the risk assessment’s aim—whether it leans toward protection or prediction.

Another aspect of CES selection is the suitability criteria to assess the BMD. The US EPA provides model suitability criteria across models, as discussed by Haber and colleagues (Haber et al., 2018). Though model averaging mitigates many of these differences, the criteria for evaluating BMD credible interval width remain relevant. Alternatives to BMDL as a reference point are recommended if the BMD is ten times lower than the lowest non-zero dose, or if the BMDU/BMDL ratio exceeds 50. As observed in Case Study 2, the CES should ideally fall within the experimental response range to avoid extrapolation, regardless of whether it lies above or below the tested dose range. When the CES is outside the observed response range, it’s essential to evaluate whether the study is suitable for deriving a PoD. However, if endpoints with biologically relevant CES are lacking, we believe that the full experimental dose set can still be applied in a sensitivity analysis. This approach helps estimate the probability that BMDL values for various preselected CES levels fall within the dose range, supporting margin of exposure calculations.

Only few studies have compared frequentist and Bayesian BMD analysis. EFSA’s BMD guidance presents two examples of Bayesian model averaging for continuous and quantal data, yielding similar results to frequentist analysis (Appendix C–Body Weight–and Appendix D–Thyroid epithelial cell vacuolization) (EFSA Scientific Committee, 2022). In our study, only Case Study 4 produced such consistent results across both approaches. Contrary to the abovementioned modeling outcomes, our results suggest that the Bayesian approach is often more effective than the frequentist method and, hence support the paradigm shift as advised by EFSA.

### 4.6 On BMD modeling of epidemiological data

Epidemiological data introduce unique challenges to BMD modeling due to high variability and potential confounding factors, as seen in Case Studies 2, 3, and 4. Case study 2 and 3 highlighted difficulties in establishing reliable BMD values, particularly when using CES above 5%. For example, in Case Study 2, the high variation in cholesterol levels led to high BMDU/BMDL ratios, and in Case Study 3, the frequentist method failed to provide stable BMD estimates at higher CES levels due to steep dose-response curves. This suggests that CES selection and model choice should be handled cautiously in epidemiological studies, as inappropriate selections can lead to unreliable or overly conservative estimates.

Overall, the use of BMD modeling for epidemiological data is challenging, with often more disperse data, when compared with, e.g., animal experiments, and loss of information, when modeling summary data instead of individual data. Especially when investigating effects from exposures to environmental pollutants, another important aspect needs appropriate consideration, that is the background exposure to compounds like PFAS can be assumed to never be zero–an issue raised by EFSA in their assessment of the Steenland study (case study 2) (EFSA CONTAM Panel, 2018).

EFSA mitigated that fact with the introduction of a baseline PFAS concentration derived from exposure data of the general European population. When considering effect sizes relevant for adversity, especially in sensitive endpoints and/or population groups, it is important to be aware of the potential of baseline exposure. Thus, exposure might already have altered the effect, influencing (potentially diminishing) effects at higher concentrations. Additionally, the cut-off for adversity becomes important in this context. The assessment whether a change in an organism can be seen as adverse or not is important for the choice of the “correct” CES in the evaluation of epidemiological data.

The US EPA’s 1SD-approach, per definition, is reliant on the variation of the control group, which might bias the remaining BMD analysis. The resulting CES and reference values could then be inadequate, simply due to the variation in the control group data, and not based on the magnitude of change observed in the dataset. The 1SD-approach proofed useful in case study 1. For all epidemiological studies, the approach did not result in informative outcomes.

As with any other approach, the different ways to estimate a useful CES present strengths and limitations. Intuitively, data-driven methods seem to outperform simpler ways of setting the CES as the mathematical definition offers an objective assessment without human bias. However, there is no one-size-fits-all effect size and, therefore, no single mathematical approach is expected to deliver the most adequate CESs.

### 4.7 Concluding remarks

Although expert judgment might be time- and resource-consuming, our study supports it as a step of fundamental importance in the selection of a CES, during a BMD analysis. The choice of CES is a matter of debate and one of the biggest challenges in the standardization of the BMD approach (Haber et al., 2018; Jensen et al., 2019). This issue is likely to persist in the future, as there appears to be no single answer which could address all possible scenarios.

The comparison of CES selections and statistical approaches across our case studies provides valuable insights into the precision and applicability of BMD modeling in regulatory assessments. The study is constrained by the limited number of comparable studies, which restricts the ability to propose broad methodological changes based on the findings. Nevertheless, we clearly observe that the choice of CES — whether a conservative 5% or a larger GTES — can dramatically influence BMDL estimates, thereby impacting conclusions on adversity thresholds. This is particularly evident when modeling variable endpoints, such as blood lipid and immune responses, where small changes may lack biological significance at the individual level but remain relevant for public health at the group level. Bayesian modeling consistently offered narrower credible intervals and proved more reliable in handling high interindividual variability. These advantages suggest that Bayesian approaches can improve upon previous EFSA and US EPA assessments by offering greater stability in estimates and by reducing overly conservative outcomes, associated with frequentist methods at low CES levels.

In practice, adopting a flexible CES that considers endpoint relevance, supported by Bayesian statistics, can offer a more transparent balance between conservatism and biological relevance. By incorporating model averaging and abandoning default CES values for endpoint-specific selections, regulatory bodies can achieve more robust and biologically justified estimates of tolerable intakes, enhancing the predictive power and applicability of risk assessments.

## Data Availability

The analyses data supporting the conclusions of this article will be made available by the authors, without undue reservation upon request.

## References

[B1] Abraham K. Mielke H. Fromme H. Volkel W. Menzel J. Peiser M. (2020). Internal exposure to perfluoroalkyl substances (PFASs) and biological markers in 101 healthy 1-year-old children: associations between levels of perfluorooctanoic acid (PFOA) and vaccine response. Arch. Toxicol. 94 (6), 2131–2147. 10.1007/s00204-020-02715-4 32227269 PMC7303054

[B2] ATSDR (2021). Toxicological profile for perfluoroalkyls. U. S. Agency Toxic Subst. Dis. Registry. 10.15620/cdc:59198 37220203

[B3] Bokkers B. G. Slob W. (2005). A comparison of ratio distributions based on the NOAEL and the benchmark approach for subchronic-to-chronic extrapolation. Toxicol. Sci. 85 (2), 1033–1040. 10.1093/toxsci/kfi144 15772368

[B4] Bokkers B. G. Slob W. (2007). Deriving a data-based interspecies assessment factor using the NOAEL and the benchmark dose approach. Crit. Rev. Toxicol. 37 (5), 355–373. 10.1080/10408440701249224 17612951

[B5] Budtz-Jorgensen E. Grandjean P. (2018). Application of benchmark analysis for mixed contaminant exposures: mutual adjustment of perfluoroalkylate substances associated with immunotoxicity. PLoS ONE 13 (10), e0205388. 10.1371/journal.pone.0205388 30339706 PMC6195268

[B6] Buist H. E. von Bolcshazy G. F. Dammann M. Telman J. Rennen M. A. (2009). Derivation of the minimal magnitude of the Critical Effect Size for continuous toxicological parameters from within-animal variation in control group data. Regul. Toxicol. Pharmacol. 55 (2), 139–150. 10.1016/j.yrtph.2009.06.009 19559065

[B7] Cordner A. De La Rosa V. Y. Schaider L. A. Rudel R. A. Richter L. Brown P. (2019). Guideline levels for PFOA and PFOS in drinking water: the role of scientific uncertainty, risk assessment decisions, and social factors. J. Expo. Sci. Environ. Epidemiol. 29 (2), 157–171. 10.1038/s41370-018-0099-9 30622333 PMC6455940

[B8] Crump K. S. (1984). A new method for determining allowable daily intakes. Fundam. Appl. Toxicol. Official J. Soc. Toxicol. 4 (5), 854–871. 10.1016/0272-0590(84)90107-6 6510615

[B9] Danish Ministry of Environment (Miljøministeriet) (2021). Bekendtgørelse om vandkvalitet og tilsyn med vandforsyningsanlæg [Notice on water quality and inspection of water supply systems] (BEK nr 2361 af 26/11/2021). Available at: https://www.retsinformation.dk/eli/lta/2021/2361.

[B10] Dekkers S. Telman J. Rennen M. A. Appel M. J. de Heer C. (2006). Within-animal variation as an indication of the minimal magnitude of the critical effect size for continuous toxicological parameters applicable in the benchmark dose approach. Risk Anal. 26 (4), 867–880. 10.1111/j.1539-6924.2006.00784.x 16948682

[B11] Dourson M. L. Hertzberg R. C. Hartung R. Blackburn K. (1985). Novel methods for the estimation of acceptable daily intake. Toxicol. Industrial Health 1 (4), 23–33. 10.1177/074823378500100404 3843503

[B12] EFSA BIOCONTAM Panel (2018). EFSA BIOCONTAM panel, UNIT on biological hazards and contaminants. Official J. Eur. Union. Article 30 of Regulation 178/2002 EFSA – ECHA – BfR - Danish EPA - RIVM, EFSA/CONTAM/3503. Available at: https://www.efsa.europa.eu/sites/default/files/news/efsa-contam-3503.pdf .

[B13] EFSA CONTAM Panel (2008). Perfluorooctane sulfonate (PFOS), perfluorooctanoic acid (PFOA) and their salts Scientific Opinion of the Panel on Contaminants in the Food chain. EFSA J. 6 (7), 653. 10.2903/j.efsa.2008.653 37213838 PMC10193653

[B14] EFSA CONTAM, Panel Knutsen H. K. Alexander J. Barregård L. Bignami M. Brüschweiler B. (2018). Risk to human health related to the presence of perfluorooctane sulfonic acid and perfluorooctanoic acid in food. EFSA J. 16 (12), e05194. 10.2903/j.efsa.2018.5194 32625773 PMC7009575

[B15] EFSA CONTAM, Panel Schrenk D. Bignami M. Bodin L. Chipman J. K. Del Mazo J. (2020). Risk to human health related to the presence of perfluoroalkyl substances in food. EFSA J. 18 (9), e06223. 10.2903/j.efsa.2020.6223 32994824 PMC7507523

[B16] EFSA Scientific Committee, Hardy A. Benford D. Halldorsson T. Jeger M. J. Knutsen K. H. (2017). Update: use of the benchmark dose approach in risk assessment. EFSA J. 15 (1), e04658. 10.2903/j.efsa.2017.4658 32625254 PMC7009819

[B17] EFSA Scientific Committee, More S. J. Bampidis V. Benford D. Bragard C. Halldorsson T. I. (2022). Guidance on the use of the benchmark dose approach in risk assessment. EFSA J. 20 (10), e07584. 10.2903/j.efsa.2022.7584 36304832 PMC9593753

[B18] European Environmental Bureau (2023). Toxic tide rising: time to tackle PFAS National approaches to address PFAS in drinking water across Europe. Brussels: E. E. Bureau. Available at: https://eeb.org/wp-content/uploads/2023/10/PFAS-in-drinking-water-briefing-final-1.pdf.

[B19] European Parliament and EU-Council (2006). Regulation (EC) No 1881/2006 setting maximum levels for certain contaminants in foodstuffs. Available at: https://eur-lex.europa.eu/legal-content/EN/LSU/?uri=CELEX%3A32006R1881&qid=1730836569943.

[B20] European Parliament and EU-Council (2020). Directive (EU) 2020/2184 of the European parliament and of the Council, 1–621 L 435. Available at: https://eur-lex.europa.eu/legal-content/EN/TXT/?uri=celex%3A32020L2184.

[B21] European Parliament and EU-Council (2023). Regulation (EU) 2023/915 on maximum levels for certain contaminants in food, Document 32023R0915. Available at: https://eur-lex.europa.eu/legal-content/EN/LSU/?uri=CELEX%3A32023R0915&qid=1730836831080.

[B22] Gaylor D. Ryan L. Krewski D. Zhu Y. (1998). Procedures for calculating benchmark doses for health risk assessment. Regul. Toxicol. Pharmacol. 28 (2), 150–164. 10.1006/rtph.1998.1247 9927564

[B23] German Federal Ministry of Health (Bundesministerium für Gesundheit) (2023). Bundesgesetzblatt Teil 1, Zweite Verordnung zur Novellierung der Trinkwasserverordnung, Nr. Available at: https://www.recht.bund.de/bgbl/1/2023/159/VO.html.

[B24] Goligher E. C. Heath A. Harhay M. O. (2024). Bayesian statistics for clinical research. Lancet 404 (10457), 1067–1076. 10.1016/S0140-6736(24)01295-9 39277290 PMC12051211

[B25] Grandjean P. Andersen E. W. Budtz-Jorgensen E. Nielsen F. Molbak K. Weihe P. (2012). Serum vaccine antibody concentrations in children exposed to perfluorinated compounds. JAMA 307 (4), 391–397. 10.1001/jama.2011.2034 22274686 PMC4402650

[B26] Haber L. T. Dourson M. L. Allen B. C. Hertzberg R. C. Parker A. Vincent M. J. (2018). Benchmark dose (BMD) modeling: current practice, issues, and challenges. Crit. Rev. Toxicol. 48 (5), 387–415. 10.1080/10408444.2018.1430121 29516780

[B27] Jensen S. M. Kluxen F. M. Ritz C. (2019). A review of recent advances in benchmark dose methodology. Risk Anal. 39 (10), 2295–2315. 10.1111/risa.13324 31046141

[B28] Kavlock R. J. Allen B. C. Faustman E. M. Kimmel C. A. (1995). Dose-response assessments for developmental toxicity: IV. Benchmark doses for fetal weight changes. Fundam. Appl. Toxicol., 26(2), 211–222. 10.1006/faat.1995.1092 7589910

[B29] Kimmel C. A. Kavlock R. J. Allen B. C. Faustman E. M. (1995). The application of benchmark dose methodology to data from prenatal developmental toxicity studies. Toxicol. Lett., 82-83, 549–554. 10.1016/0378-4274(95)03500-1 8597108

[B30] Kremer C. O. O. Shkedy Z. Aerts M. Verlinden W. Varewyck M. Verbeke T. (2022). Bayesian Benchmark Dose Model. WEB app. Zenodo. (Version 4). 10.5281/zenodo.7986184

[B31] Leisenring W. Ryan L. (1992). Statistical properties of the NOAEL. Regul. Toxicol. Pharmacol. 15 (2 Pt 1), 161–171. 10.1016/0273-2300(92)90047-d 1626067

[B32] Lewington S. Whitlock G. Clarke R. Sherliker P. Emberson J. Halsey J. (2007). Blood cholesterol and vascular mortality by age, sex, and blood pressure: a meta-analysis of individual data from 61 prospective studies with 55,000 vascular deaths. Lancet 370 (9602), 1829–1839. 10.1016/s0140-6736(07)61778-4 18061058

[B33] Livsmedelsverket (Swedish Food Agency) (2022). Livsmedelsverkets föreskrifter om dricksvatten, LIVSFS 12, Available at: https://www.livsmedelsverket.se/globalassets/om-oss/lagstiftning/dricksvatten---naturl-mineralv---kallv/livsfs-2022-12_web_t.pdf

[B34] Mihaylova B. Emberson J. Blackwell L. Keech A. Simes J. Barnes E. (2012). The effects of lowering LDL cholesterol with statin therapy in people at low risk of vascular disease: meta-analysis of individual data from 27 randomised trials. Lancet, 380(9841), 581–590. 10.1016/S0140-6736(12)60367-5 22607822 PMC3437972

[B35] Ministerio de la presidencia Relaciones Con Las C. Y. Memoria D. (2023). BOE-A-2023-628 Royal Decree 3/2023, of January 10, technical-sanitary criteria for the quality of drinking water, its control and supply, Real Decreto 3/2023, de 10 de enero, por el que se establecen los criterios técnico-sanitarios de la calidad del agua de consumo, su control y suministro. Available at: https://www.boe.es/boe/dias/2023/01/11/pdfs/BOE-A-2023-628.pdf.

[B36] Moerbeek M. Piersma A. H. Slob W. (2004). A comparison of three methods for calculating confidence intervals for the benchmark dose. Risk Anal. 24 (1), 31–40. 10.1111/j.0272-4332.2004.00409.x 15027998

[B37] Öberg M. Palmen N. Johanson G. (2010). Discrepancy among acute guideline levels for emergency response. J. Hazard Mater 184 (1-3), 439–447. 10.1016/j.jhazmat.2010.08.054 20851517

[B38] OECD. (2021). Reconciling terminology of the universe of per- and polyfluoroalkyl substances. 10.1787/e458e796-en

[B39] Piepoli M. F. Hoes A. W. Agewall S. Albus C. Brotons C. Catapano A. L. (2016). 2016 European guidelines on cardiovascular disease prevention in clinical practice: the sixth joint task force of the European society of cardiology and other societies on cardiovascular disease prevention in clinical practice (constituted by representatives of 10 societies and by invited experts)Developed with the special contribution of the European association for cardiovascular prevention and rehabilitation (EACPR). Eur. Heart J. 37 (29), 2315–2381. 10.1093/eurheartj/ehw106 27222591 PMC4986030

[B40] Reinikainen J. Bouhoulle E. Sorvari J. (2024). Inconsistencies in the EU regulatory risk assessment of PFAS call for readjustment. Environ. Int. 186, 108614. 10.1016/j.envint.2024.108614 38583295

[B41] Ringblom J. Johanson G. Oberg M. (2014). Current modeling practice may lead to falsely high benchmark dose estimates. Regul. Toxicol. Pharmacol. 69 (2), 171–177. 10.1016/j.yrtph.2014.03.004 24662478

[B42] RIVM (2022). Rijksinstituut voor Volksgezondheid en Milieu, PFAS in Nederlands drinkwater vergeleken met de nieuwe Europese Drinkwaterrichtlijn en relatie met gezondheidskundige grenswaarde van EFSA. Available at: https://www.rivm.nl/bibliotheek/rapporten/2022-0149.pdf.

[B43] Sand S. Filipsson A. F. Victorin K. (2002). Evaluation of the benchmark dose method for dichotomous data: model dependence and model selection. Regul. Toxicol. Pharmacol., 36(2), 184–197. 10.1006/rtph.2002.1578 12460753

[B44] Sand S. Victorin K. Filipsson A. F. (2008). The current state of knowledge on the use of the benchmark dose concept in risk assessment. J. Appl. Toxicol. 28 (4), 405–421. 10.1002/jat.1298 17879232

[B45] Schenk L. Ho M. R. Taxell P. Huuskonen P. Leite M. Martinsone I. (2024). Occupational exposure limits for reproductive toxicants - a comparative analysis. Reprod. Toxicol. 128, 108649. 10.1016/j.reprotox.2024.108649 38942216

[B46] Seacat A. M. Thomford P. J. Hansen K. J. Olsen G. W. Case M. T. Butenhoff J. L. (2002). Subchronic toxicity studies on perfluorooctanesulfonate potassium salt in cynomolgus monkeys. Toxicol. Sci. 68 (1), 249–264. 10.1093/toxsci/68.1.249 12075127

[B47] Setzer R. W. Kimmel C. A. (2003). Use of NOAEL, benchmark dose, and other models for human risk assessment of hormonally active substances. Pure Appl. Chem. 75 (11-12), 2151–2158. 10.1351/pac200375112151

[B48] Shao K. Shapiro A. J. (2018). A web-based system for bayesian benchmark dose estimation. Environ. Health Perspect. 126 (1), 017002. 10.1289/EHP1289 29329100 PMC6014690

[B49] Slob W. (2002). Dose-response modeling of continuous endpoints. Toxicol. Sci. 66 (2), 298–312. 10.1093/toxsci/66.2.298 11896297

[B50] Slob W. (2014). Benchmark dose and the three Rs. Part I. Getting more information from the same number of animals. Crit. Rev. Toxicol. 44 (7), 557–567. 10.3109/10408444.2014.925423 25000332

[B51] Slob W. (2017). A general theory of effect size, and its consequences for defining the benchmark response (BMR) for continuous endpoints. Crit. Rev. Toxicol. 47 (4), 342–351. 10.1080/10408444.2016.1241756 27805866

[B53] Steenland K. Tinker S. Frisbee S. Ducatman A. Vaccarino V. (2009). Association of perfluorooctanoic acid and perfluorooctane sulfonate with serum lipids among adults living near a chemical plant. Am. J. Epidemiol. 170 (10), 1268–1278. 10.1093/aje/kwp279 19846564

[B54] US EPA. (1995). United States environmental protection agency, the use of the benchmark dose approach in health risk assessment. In.

[B55] US-EPA (2012a). Benchmark dose technical guidance. (EPA/100/R-12/001). Washington: Risk Assessment Forum U.S. Environmental Protection Agency. Available at: https://www.epa.gov/risk/benchmark-dose-technical-guidance.

[B56] US-EPA (2012b). Benchmark dose technical guidance. (EPA/100/R-12/001). Washington, DC: Risk Assessment Forum U.S. Environmental Protection Agency. Available at: https://www.epa.gov/risk/benchmark-dose-technical-guidance.

[B57] US EPA. (2022a). United States environmental protection agency, INTERIM drinking water health advisory: perfluorooctane sulfonic acid (PFOS) CASRN 1763-23-1. In.

[B58] US EPA. (2022b). United States environmental protection agency, science advisory board, transmittal of the science advisory board report titled, “review of EPA’s analyses to support EPA's national primary drinking water rulemaking for PFAS”, EPA-SAB-22-008. In.

[B59] Varewyck M. Verbeke T. (2019). Software for benchmark dose modelling. EFSA Support. Publ. 16 (1). 10.2903/sp.efsa.2019.en-1489

[B60] Vieira Silva A. Ringblom J. Moldeus P. Tornqvist E. Oberg M. (2021). Benchmark dose-response analyses for multiple endpoints in drug safety evaluation. Toxicol. Appl. Pharmacol. 433, 115732. 10.1016/j.taap.2021.115732 34606779

[B61] Vrancken K. (2022). Executive summary final report closing the circle? Available at: https://www.vlaanderen.be/en/pfas-in-flanders/final-report-on-the-pfas-contamination#summary-of-final-report-on-the-pfas-contamination.

[B62] Wheeler M. W. Bailer A. J. (2007). Properties of model-averaged BMDLs: a study of model averaging in dichotomous response risk estimation. Risk Anal. 27 (3), 659–670. 10.1111/j.1539-6924.2007.00920.x 17640214

[B63] Wheeler M. W. Bailer A. J. (2008a). Comparing model averaging with other model selection strategies for benchmark dose estimation. Environ. Ecol. Statistics 16 (1), 37–51. 10.1007/s10651-007-0071-7

[B64] Wheeler M. W. Bailer A. J. (2008b). Model averaging software for dichotomous dose response risk estimation. J. Stat. Softw. 26 (5), 1–15. 10.18637/jss.v026.i05 19777145

[B65] Wheeler M. W. Bailer A. J. (2009). Benchmark dose estimation incorporating multiple data sources. Risk Anal. 29 (2), 249–256. 10.1111/j.1539-6924.2008.01144.x 19000080

[B66] WHO (2009). World health organization, food, agriculture organization of the united, nations principles and methods for the risk assessment of chemicals in food environmental health criteria, 240. Geneva: World Health Organization. EHC 240.

[B67] WHO. (2017). WHO, World Health Organization, Diphtheria vaccine Review of evidence on vaccine effectiveness and immunogenicity to assess the duration of protection ≥10 years after the last booster dose. In.

[B68] WHO. (2020). World health organization, food, agriculture organization of the united, nations principles and methods for the risk assessment of chemicals in food, Second edition, Environmental Health Criteria 240 (EHC 240), Amended Chapter 5, Dose–response assessment and derivation of health-based guidance values. In. Geneva: World Health Organization.

[B69] WHO. (2021). Human health risk assessment toolkit: chemical hazards, second edition, Geneva: World Health Organization. Available at: https://www.who.int/publications/i/item/9789240035720 , Accessed 03.December.2022.

[B70] Zarn J. A. Hanggi E. Engeli B. E. (2015). Impact of study design and database parameters on NOAEL distributions used for toxicological concern (TTC) values. Regul. Toxicol. Pharmacol. 72 (3), 491–500. 10.1016/j.yrtph.2015.05.015 26001586

