# Supplementary material for: Selection of the critical effect size alters hazard characterization – a retrospective analysis of key studies used for risk assessments of PFAS
Source: Front Toxicol. 2025 Mar 14;7:1525089. doi: 10.3389/ftox.2025.1525089 (PMC11949891; doi:10.3389/ftox.2025.1525089)
Supplement: Supplementary file 1 [file DataSheet1.pdf]

Supplement 2:

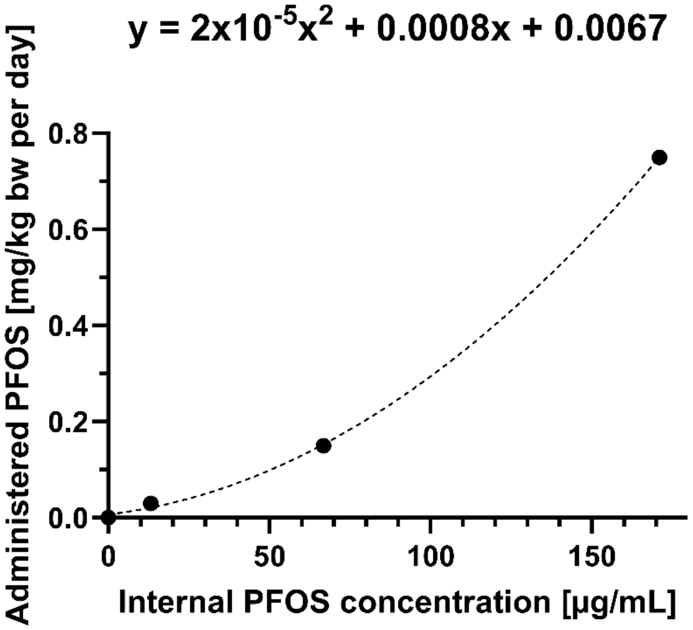

Supplementary Figure 1: Second order polynomial (quadratic) function describing the administered doses of potassium PFOS (y-axis) and the corresponding serum PFOS concentrations (x-axis) in female Cynomolgus Monkeys measured after 183 days of treatment, as described in Seacat et al. (2002). This function was used to back calculate the theoretically administered doses departing from the BMD estimates obtained by modelling the internal PFOS concentrations, as described in the manuscript.
